# Effects of the Higenamine, a Potent Compound from *Aconitum*, on UVB-Induced Photoaging in Hairless Mice

**DOI:** 10.1155/2022/9116642

**Published:** 2022-04-27

**Authors:** Hye-Sun Lim, Yumi Jang, Gunhyuk Park

**Affiliations:** Herbal Medicine Resources Research Center, Korea Institute of Oriental Medicine, 111 Geonjae-ro, Naju-si, Jeollanam-do 58245, Republic of Korea

## Abstract

**Aim:**

Higenamine [1-[(4-hydroxyphenyl) methyl]-1, 2, 3, 4-tetrahydroisoquinoline-6, 7-diol], a potent cardiotonic compound from *Aconitum*, contributes to vascular relaxation and bronchodilation. However, the effects and mechanisms of action of higenamine on skin aging remain poorly understood. In this study, the effects of higenamine on UVB-induced photoaging were examined in the hairless mouse model.

**Methods:**

The dorsal skin of hairless mice (CrlOri : SKH1) was exposed to chronic UVB irradiation (100–300 mJ/cm^2^ for 6 weeks), with subsequent administration of higenamine (1–20 mg/kg, p.o.) for 2 weeks. TGF-*β*, Smad3 DNA-binding phosphorylation, and COL1A1 levels were analyzed by immunohistochemistry, and histological analysis of the skin was performed via H&E and MT staining.

**Results:**

Higenamine increased TGF-*β*, Smad3 DNA-binding phosphorylation, and COL1A1 expression in primary human fibroblast cells and mouse skin. Higenamine suppressed UVB-induced photoaging via skin recovery, improved epidermal thickness, and prevented Smad3, DNA-binding phosphorylation, and COL1A1 depletion via TGF-*β* signaling.

**Conclusion:**

Higenamine enhances collagen production in the skin through TGF-*β*/Smad3 signaling and potentially suppresses UVB-induced skin aging.

## 1. Introduction

Skin aging is classified into intrinsic aging, a natural consequence of physiological changes over time, and extrinsic aging, which involves various accelerating factors, including pollutants, smoking, and ultraviolet (UV) irradiation [1, 2]. Notably, the main cause of skin aging is UV irradiation characterized by hyperpigmentation, sagging, and skin wrinkles [3]. UV irradiation increases the level of phosphorylation of mitogen-activated protein kinases (MAPKs) and, most importantly, the production of reactive oxygen species [3]. MAPK regulates activator protein 1 (AP-1), which facilitates matrix metalloproteinase (MMP) transcription, breaking down major components of the extracellular matrix (ECM), including elastin, proteoglycan, and collagen [4]. Transforming growth factor beta (TGF-*β*) promotes the synthesis and secretion of elastin and collagen and downregulates the enzymes involved in collagen decomposition [4, 5]. Particularly, TGF-*β*-induced signals are upregulated by Smad2/3 and downregulated by Smad7 [5, 6]. Therefore, regulation of the TGF-*β*/Smad pathway is crucial in maintaining skin health and repairing skin damage.

**Figure 1:**
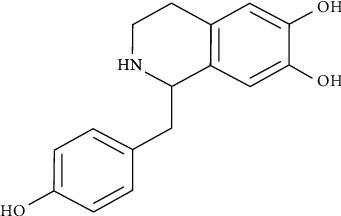
Chemical structure of higenamine.

Higenamine [1-[(4-hydroxyphenyl) methyl]-1, 2, 3, 4-tetrahydroisoquinoline-6, 7-diol] (Figure 1), chemically called dl-demethylcoclaurine, is a monobenzylisoquinoline alkaloid member of the protoberberine class of compounds [7]. Higenamine is extracted from various species of plants from the genus, *Aconitum*, such as *Aconitum carmichaelii*, *Aconitum kusnezoffii*, and *Aconitum jaluense*; it is a potent cardiotonic. *Aconitum carmichaelii* is used as an oriental medicine in Korea, China, and Japan for the treatment of diverse health conditions [7]. Its utility in heart attack, troubled breathing, arthritis, shortness of breath, sepsis, arrhythmia, heart failure, and erectile dysfunction was reported recently [8, 9]. Higenamine modulates adrenoceptors, inducing a positive ionotropic effect [10]. It also exhibits an antithrombotic effect, inhibits platelet aggregation via a cAMP-dependent pathway, and activates radical scavenging activity and heme oxygenase-1 [11]. Furthermore, it is predicted to have antiapoptotic and inflammatory effects including inhibiting PI3K/AKT signal modulation, reducing NF-*κ*B, and inhibiting the mitochondrial-related apoptosis signaling pathway [12, 13]. It also induces cardiac fibrosis by blocking the TGF-*β*/Smad signaling pathway. However, the pharmacological and physiological effects of higenamine on UVB-mediated photoaging are not well understood. In this study, we investigated higenamine effects on COL1A1 induction and its inhibitory effect on UVB-induced photoaging and skin lesions in hairless mice. We also explored the possible mechanisms underlying the pharmacological effects of higenamine by assessing the response of the TGF-*β*/Smad signaling pathway.

## 2. Materials and Methods

### 2.1. Chemicals and Reagents

Dulbecco's modified Eagle's medium (DMEM), penicillin-streptomycin, fetal bovine serum (FBS), and Opti-MEM were purchased from Gibco (Waltham, MA, USA) and Lipofectamine 2000 from Invitrogen (Carlsbad, CA, USA). MTT, higenamine, hematoxylin-eosin (H&E) staining solution, and dimethyl sulfoxide (DMSO) were purchased from Sigma-Aldrich (St. Louis, USA). The Trichrome stain kit was purchased from Abcam (Cambridge, UK). Smad3 DNA-binding phosphorylation and TGF-*β* ELISA kits were obtained from LSBio (Seattle, WA, USA). The COL1A1 ELISA kit was obtained from Cusabio Technology (Wuhan, China). All other reagents belonged to a guaranteed or an analytical grade.

### 2.2. Animals and UVB Irradiation

Female hairless mice (CrlOri : SKH1, 5 weeks old) were purchased from the Orient Experimental Animal Breeding Center (Seoul, Korea). The animals were maintained under temperature and light-controlled conditions (23 ± 1°C, 12 h light/dark cycle) with food and water provided ad libitum. The experimental protocol and design of the study were approved by the Committee on Animal Care of the Korea Institute of Oriental Medicine (KIOM; approval no. KIOM-20-071 and KIOM-21-021). Mice were distributed (*n*) among two separate experimental sets, *A* and *B*, which comprised nine groups: A1, control (*n* = 7); A2, higenamine 1 mg/kg (*n* = 7); A3, higenamine 5 mg/kg (*n* = 7); A4, higenamine 10 mg/kg (*n* = 7); A5, higenamine 20 mg/kg (*n* = 7); B1, control (*n* = 7); B2, UVB (*n* = 7); B3, UVB + higenamine 5 mg/kg (*n* = 7); B4, UVB + higenamine 10 mg/kg (*n* = 7); and B5, UVB +  higenamine 20 mg/kg (*n* = 7).

UVB irradiation was performed by exposing the dorsal skin of anesthetized mice to chronic excessive UVB irradiation, i.e., 100–300 mJ/cm^2^ for 3 days per week (Monday, Wednesday, and Friday) for 6 weeks. Then, higenamine dissolved in normal saline was administered for 7 days per week, p.o., for 2 weeks to specific treatment groups.

### 2.3. Histological Examination Using Hematoxylin and Eosin (H&E) and Masson's Trichrome (Mt) Staining

The dorsal skin of each mouse was removed and fixed in 10% formalin. Serial sections of 4 *μ*m thickness were obtained using a paraffin microtome designed for histological analysis using H&E and MT staining kits.

### 2.4. Measurement of Transepidermal Water Loss

Transepidermal water loss (TEWL) was used as a permeability marker to indicate skin barrier function and was measured using the Tewameter® TM300 (Courage + Khazaka Electronic GmbH, Cologne, Germany) 10 weeks after the experiment started. Values were recorded once the responses stabilized, typically 10 s after placing the probe on the skin. TEWL was measured as the average of three iterations in each test area.

### 2.5. Cell Culture

The cell culture system was established as per previous reports [14, 15]. Human primary dermal fibroblast cells were purchased from TEGO Science (Seoul, Korea) and maintained in a fibroblast medium containing fibroblast growth supplement, 10% heat-inactivated FBS, and 1% penicillin-streptomycin. All experiments were conducted 12 h after the cells had been seeded on the 96- and 24-well plates at densities of 1 × 10^4^ and 2 × 10^4^ cells/well, respectively.

### 2.6. Measurement of Cytotoxicity

Cells were treated simultaneously with higenamine (0.01–10 *μ*M) for 24 h and then incubated with 1 mg/mL MTT for 3 h. The medium was aspirated from the wells, and the formazan dye was dissolved in DMSO. The absorbance was measured using a spectrophotometer (VersaMax microplate reader, Molecular Device, Sunnyvale, CA, USA) at a wavelength of 570 nm and expressed as a percentage of the control value.

### 2.7. Transfection of Small Interfering RNA

Human primary dermal fibroblast cells were used at a confluence of 80–85% in 100 mm dishes. Cells were transfected with stealth small interfering RNA (siRNA) using Lipofectamine 2000 (5 *μ*L) mixed with 30 *μ*M siRNA solution (an equimolar mix of TGF-*β* siRNA and scrambled siRNA) and 3 mL of Opti-MEM (Gibco). After incubation for 30 min at room temperature, the mix (500 *μ*L) was added into serum-free DMEM (500 *μ*L) in each dish and incubated for 12 h.

### 2.8. Statistical Analysis

The values are expressed as mean ± standard error of the mean (SEM). The statistical variables were analyzed using one-way analysis of variance (ANOVA) post hoc multiple mean comparisons (Tukey's HSD test). Statistical significance was set at *p* < 0.05. All variables were analyzed using the GraphPad Prism 5.10 software (GraphPad Software Inc., San Diego, CA, USA).

## 3. Results

### 3.1. Upregulation of Collagen and Associated Signaling by Higenamine In Vivo

**Figure 2:**
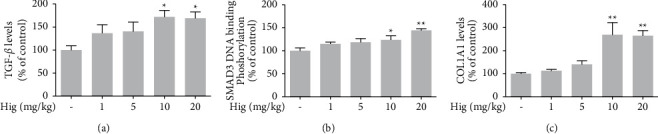
Promotive effect of higenamine on the collagen-related proteins. The levels of TGF-*β* (a), Smad3 (b), and COL1A1 (c) determined by ELISA. Values are expressed as the mean ± standard error of the mean. ^*∗*^*P* < 0.05 and ^*∗∗*^*P* < 0.01 compared to the control group.

To investigate the effects of higenamine on collagen regulation, we determined the COL1A1 levels and its associated signaling pathway genes in the skin of hairless mice. ELISA revealed increased levels of COL1A1, TGF-*β*, and Smad3 DNA-binding phosphorylation in nuclear fractions upon higenamine (1–20 mg/kg) treatment (Figures 2(a)–2(c)).

### 3.2. Effects of Higenamine on UVB-Induced Skin Abnormalities In Vivo

**Figure 3:**
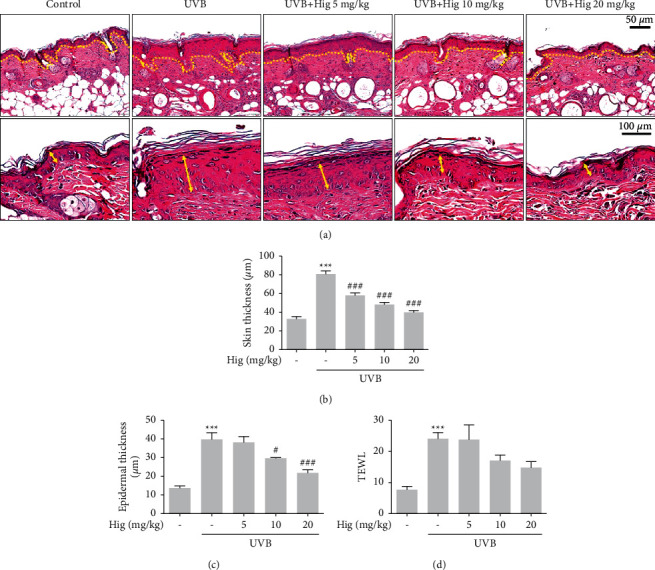
Effect of higenamine on UVB-induced photoaging in the skin of mice. Representative histological analysis of skin section damaged by UVB exposure. (a)–(c) Hematoxylin/eosin staining used to identify epidermal thickness. TEWL measured using a Tewameter (d). Values are expressed as the mean ± standard error of the mean. ^###^*P* < 0.001 compared to the control group, and ^#^*P* < 0.05 and ^###^*P* < 0.001 compared to the UVB-alone group.

To determine the effects of higenamine on photoaging, such as skin lesions induced by UVB, we measured the skin thickness, epidermal thickness, and TEWL, along with the histological assessment. UVB irradiation significantly increased skin and epidermal thickness, which reduced after treatment with 5–20 mg/kg higenamine (Figure 3). However, the level of TEWL tended to decrease with higenamine treatment; however, the decrease was not statistically significant (Figure 3).

### 3.3. Effects of Higenamine on UVB-Induced Modulations in Collagen Structure and Levels and Related Signaling

**Figure 4:**
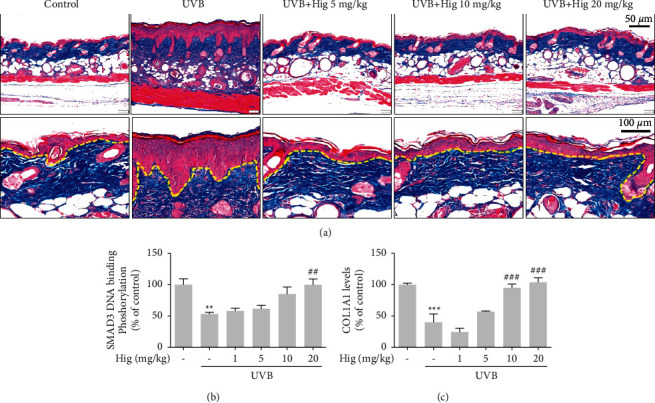
Effect of higenamine on UVB-induced photoaging in the skin of mice. Representative histological analysis of skin section damaged by UVB exposure. (a) Masson's trichrome staining to identify collagen fibers. The levels of Smad3 (b) and COL1A1 (c) determined by ELISA. Values are expressed as the mean ± standard error of the mean. ^*∗∗*^*P* < 0.01 and ^*∗∗∗*^*P* < 0.001 compared to the control group, and ^##^*P* < 0.01 and ^###^*P* < 0.001 compared to the UVB-alone group.

To determine whether higenamine affects collagen and associated cytokines, we determined the MT histology and levels of Smad3 and COL1A1 in mouse skin. Irradiation with UVB significantly decreased collagen fiber levels. This effect was reduced on treatment with 5–20 mg/kg higenamine (Figure 4(a)). Additionally, UVB irradiation significantly decreased Smad3 DNA-binding phosphorylation and COL1A1 levels, which were increased by higenamine (5–20 mg/kg treatment) (Figures 4(b) and 4(c)).

### 3.4. Effect of si-TGF-*β* against Higenamine on Collagen following UVB Irradiation in Primary Human Skin Fibroblast Cells

**Figure 5:**
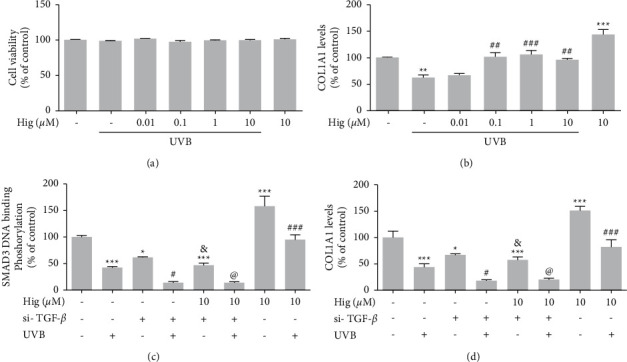
Effect of higenamine on the levels of UVB-induced photoaging in vitro. Cytotoxicity levels measured by MTT (a), and COL1A1 levels measured by ELISA (b) in cells treated with higenamine followed by UVB stimulation. Levels of Smad2 DNA-binding Phosphorylation (c) and COL1A1 (d) in TGF-*β* siRNA-transfected differentiated human primary fibroblast cells. Values are expressed as the mean ± standard error of the mean. ^#^*P* < 0.05 and ^###^*P* < 0.001 compared to the control group, ^*∗*^*P* < 0.05, ^*∗∗*^*P* < 0.01, and ^*∗∗∗*^*P* < 0.001 compared to the UVB-alone group, ^&^*P* < 0.05 compared to the si-TGF-*β* group, and ^@^*P* < 0.05 compared to the higenamine + si-TGF-*β* + UVB group.

The MTT assay revealed no effect of higenamine treatment (0.01–10 *μ*M) on the viability of human primary dermal fibroblast cells (Figure 5(a)). Moreover, UVB irradiation significantly decreased COL1A1 levels compared to that in control, while treatment with 0.01–10 *μ*M higenamine increased UVB-induced COL1A1 expression (Figure 5(b)). To assess the importance of TGF-*β* in UVB irradiation-induced damage and to compare the effects of TGF-*β* regulators with that of higenamine, Smad3 DNA-binding phosphorylation and COL1A1 levels were analyzed in TGF-*β* knockdown human primary dermal fibroblast cells. In TGF-*β* siRNA-transfected cells, the levels of both proteins declined significantly by UVB irradiation, whereas remained unaffected by UVB irradiation when the cells were treated with 10 *μ*M higenamine (Figures 5(c) and 5(d), as compared to control TGF-*β* siRNA-transfected cells.

## 4. Discussion

The dermis is predominantly composed of collagen, along with elastic fibers, nerves, blood vessels, hair follicles, and secretory glands [16]. The main cell types in the dermis are fibroblasts, vascular smooth muscle cells, macrophages, fat cells, adipocytes, Schwann cells, and follicle stem cells [16]. Fibroblasts provide a collagen-rich extracellular matrix (ECM), while the blood vessels and lymphatic vessels maintain the immune cell infiltration [16]. A photoaged dermis is characterized by reduced thickness, decreased collagen content, poor organization, fragmented collagen fibers, elastic fiber degradation, and severe dermal connective tissue damage [17, 18]. Such changes result in the most visible signs of skin aging, including wrinkle formation, fragile atrophic skin, delayed wound healing, and sagging [18].

Damaged collagen fibrils and elastin fibers are the most evident features of a dermis aged from UVB exposure, mediated by MMP synthesis via MAPK signaling [19, 20]. MMPs are a family of ubiquitous endopeptidases involved in the inflammatory response by regulating chemokine activity [21]. MMPs are responsible for complete collagen degradation through collagenase-1 (MMP-1), stromelysin-1 (MMP-3), and gelatinase B (MMP-9) [21]. Furthermore, UVB causes collagen degradation by downregulating the TGF-/Smad signaling pathway resulting in reduced procollagen synthesis [21]. TGF-*β* primarily transfers signals by binding to cell surface receptors (TGF-*β* type I and type II receptors) to phosphorylate the Smad2/Smad3 complex and activate the promoter of COL1A2 through Smad signal transmission [22, 23]. UVB irradiation inhibits Smad2/Smad3 nuclear translocation, resulting in decreased expression of TGF-*β* type II receptor and procollagen type I in hairless mice [22].

**Figure 6:**
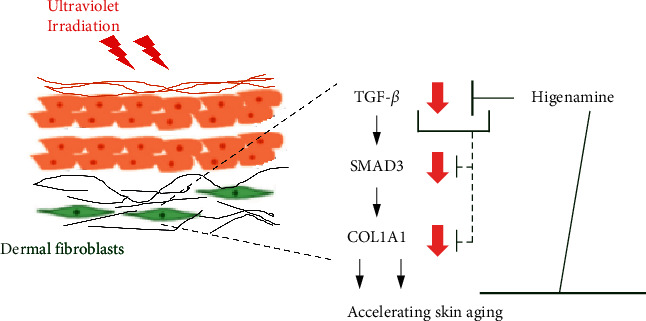
Schematic of the mechanism proposed for the higenamine effects on UVB-induced photoaging via upregulation of TGF-*β*/Smad3 in hairless mice.

In this study, after screening numerous compounds that might influence the relationship between collagen and Smad, we selected higenamine derived from *Aconitum japonicum*. We first investigated the effect of higenamine on TGF-*β*, COL1A1, and Smad3 DNA-binding phosphorylation levels in the skin of mice, which showed a significant increase. Therefore, we administered higenamine in a mouse model with UVB-induced skin aging, where recovery of increased skin thickness and reduced collagen levels was noted. In addition, the expression of suppressed Smad3 was significantly increased. Furthermore, since the TGF-*β* signal is a key in UVB-induced damage, we developed TGF-*β* knockdown models using siRNA-transfected human primary fibroblast cells to assess whether higenamine affects TGF-*β* expression. Consequently, we confirmed that higenamine does not affect Smad3 and collagen precursor genes and significantly contributes in skin collagen production through TGF-*β* expression (Figure 6).

This study has some limitations. First, there is lack of information on the transport of higenamine to other parts when applied to the skin. Its effect is known; however, the exact dose and time for ensuring optimal efficacy is unclear. In addition, understanding the effects and efficiency when developed as emulsion-type cosmetics or external application agents warrants additional research. Second, these results should be verified using an animal model in which TGF-*β* is suppressed. Finally, it is important to evaluate the efficacy of higenamine in human studies through clinical application tests; this would enable the development of antiaging and enhancement of skin agents.

Overall, we demonstrate that higenamine can improve collagen production in the skin through TGF-*β*/Smad3 signaling and suppress UVB-induced skin aging in mice models. Although several studies support the role of higenamine in suppression of skin aging, further research using TGF gene-deficient mice and actual clinical studies are required to confirm these effects considering the potential use of higenamine in cosmetics and health functional foods.

## Data Availability

The data used to support the findings of this study are available from the corresponding author upon request.
